# Knowledge and Attitude of Teenagers Towards Electronic Cigarettes in Karachi, Pakistan

**DOI:** 10.7759/cureus.1468

**Published:** 2017-07-13

**Authors:** Asim Shaikh, Hamza T Ansari, Zeerak Ahmad, Mahnoor Y Shaikh, Ilma Khalid, Maha Jahangir, Amna Majeed, Nimra Shakeel, Arsalan Ahmed, Roha Saeed Memon, Eleze Tariq, Rafia Irfan, Dania Madni

**Affiliations:** 1 Dow Medical College, Dow University of Health Sciences (DUHS), Karachi, Pakistan; 2 Ziauddin Medical College, Ziauddin University; 3 Aga Khan University, The Aga Khan University; 4 Ziauddin University

**Keywords:** electronic, cigarettes, pakistan, knowledge, attitude, smoking, e-cigarettes, karachi, teenagers, nicotine

## Abstract

Introduction

Studies have shown that electronic cigarettes have gained immense popularity and their use has increased dramatically all over the world. However, little is known about the knowledge and attitudes towards e-cigarettes in third world countries such as Pakistan. The aim of this study was to determine the perceptions of teenagers in Karachi regarding e-cigarettes and whether the differences in said perceptions were affected by gender and level of education.

Methods

We conducted a cross-sectional study in January 2017 using convenience sampling and interviewed 441 young individuals, aged 13 to 19 years, to determine their knowledge, attitudes, and practices regarding e-cigarettes. The participants were questioned about their knowledge and its source. Attitudes were judged using four and five-point Likert scales while questions regarding practices focused on single and current use. Chi-square and Mann-Whitney tests were applied to compare the knowledge, attitudes, and practices of teenagers with gender and level of schooling.

Results

The majority of participants knew what e-cigarettes were (n=277, 68.7%) but did not know about their contents (n=225, 55.8%) and had learned about them from either friends or the internet (n=245, 60.%). Almost half of them (n=190, 47.2%) believed that the reason for e-cigarette use was either peer pressure or to quit smoking conventional cigarettes. An overwhelming majority also stated that; it was either easy or very easy to obtain e-cigarettes (n=277, 68.7%), they would not try smoking e-cigarettes even if a good friend of theirs recommended them (n=287, 71.2%), they were not current e-cigarette smokers (n=370, 91.8%) and they would never promote e-cigarette use (n=371, 92.1%). Statistically significant differences were found with males knowing more about e-cigarettes (p=0.006) and being more common to either have smoked (p <0.001) or be current e-cigarette smokers (p <0.001). Furthermore, middle school students were more likely to have negative attitudes towards e-cigarettes believing they were more harmful (p=0.003) and more addictive (p <0.001) than conventional cigarettes.

Conclusion

Many people were aware of what electronic cigarettes are but still, it was evident that there was the lack of proper knowledge along with negative attitudes towards e-cigarette use among teenagers in Pakistan due to cultural and social stigmas and lack of advertising. Males and females had considerable differences in their opinions regarding e-cigarette use owing to such social practices being considered taboo by females and males having greater freedom due to patriarchal, familial and cultural systems.

## Introduction

The surge in the use of tobacco imposes a huge worry on public health and it has been linked to significant increases in chances of incurring coronary heart diseases, strokes and lung cancers [1]. An example of nicotine’s extremely addictive nature is that 80% of the smokers in the United States fail in their attempt to quit smoking on their own [2]. This has been one of the major reasons that have led to the development of electronic cigarettes. E-cigarettes are machines that contain a vaporizer to produce nicotine infused smoke that provides the user with an effect similar to that of conventional cigarettes [3] and it has become one of the most common methods of managing nicotine addiction without the use of tobacco.

This invention has grabbed the attention of many, compelling an increasing number of the youth to try it [4]. A tremendous increase in awareness and use of e-cigarettes has been witnessed amongst the youth [4]. Teenagers in schools have also begun using e-cigarettes [5-6]. Studies conducted in the United States to find the prevalence of e-cigarette consumption have found an increase of 6.8% in lifetime use prevalence in young adults in 2012 as compared to 2011 [7]. Another study [8] concluded that the consumption of e-cigarettes and conventional cigarettes simultaneously had doubled in the United States between 2011 and 2012. However, the effect of e-cigarette smoking on public health is still unknown [9]. In addition to being unapproved by the Food and Drug Administration (FDA) [10], the existence of 466 brands and 7764 flavors [11] complicates the assessment of future health risks or benefits. Although substantial amounts of studies have been conducted regarding the topic of e-cigarettes and their role in the lives of teenagers, there is a lack of data in different parts of the world, such as Pakistan, regarding the perceptions and knowledge of teenagers towards e-cigarettes.

This study focused, primarily, on the attitudes and knowledge of teenagers towards e-cigarettes in Karachi, Pakistan, while having a secondary focus on teenagers’ practices regarding e-cigarettes.

## Materials and methods

In January 2017, we conducted a four-week cross-sectional study to determine the attitudes and knowledge of teenagers in Karachi towards e-cigarettes after approval from the institutional review board of Dow University of Health Sciences. The sample size of 384 was calculated using a confidence level of 95% and a frequency of outcome factor of 50%. We interviewed 441 young individuals using convenience sampling, out of which 23 (5.2%) refused to take part in the study and a further 15 (3.6%) did not fill out the questionnaire completely. Thus, the cooperation rate was 91.4%. Anonymity was ensured and written consent was taken. For people under 18, the parent’s written consent was taken. All individuals with ages ranging from 13 to 19 were allowed to participate in the study. No socioeconomic restrictions were placed such as household income and enrollment in schools. The only restrictions placed were age and city of residence which had to be Karachi. The questionnaire was initially written in English and translated into Urdu for people who could not read English. Three groups of two people each conducted face-to-face interviews while using a pre-coded questionnaire. All interviewers were ordered to wear identical lab coats, offer prepared explanations for questions, not to engage in mundane conversations and offer the same amount of time to each person. This approach aimed to reduce interviewer and instruction bias. Complete anonymity was ensured to minimize response bias and a small number of relatively recent practice questions, up to one month, was asked to limit recall bias.

The questionnaire consisted of a total of 21 questions and was divided into three parts. The first part focused on questions regarding the knowledge of e-cigarettes among participants while the second and third parts of the questionnaire focused mainly on the attitude and practices regarding e-cigarettes among individuals respectively. Questions regarding the assessment of knowledge were whether the participants knew about e-cigarettes, their ingredients, and levels of Nicotine (0 mg, 6 mg, 12 mg, and 18 mg) and the source of this knowledge. The attitude of participants toward e-cigarettes was assessed by using four and five-point Likert scales. Questions regarding the comparison of the effects and addiction of e-cigarettes with normal cigarettes were also asked. The practice questions mainly focused on the use of e-cigarettes once or current smoking in the past 30 days.

Data were entered and analyzed using Statistical Package for the Social Sciences (SPSS) version 23.0 (IBM Corp., Armonk, New York). Frequencies and percentages were computed for categorical responses. Chi-square test and Man-Whitney test were applied to correlate gender with knowledge, perceptions, and practices of teenagers towards e-cigarettes.

## Results

Out of the 403 individuals who completed the questionnaire, the majority (n=256, 63.5%) were females. The mean age of the individuals was 16.4± 2.3 years with, about one-third (n=130, 32.3%) being 19 years old and more than half of them (n=212, 52.6%) being enrolled at high schools. About one tenth of the group (n=49, 12.2%), were not enrolled in any schools and almost all of them (n=41, 10.2%) were 19 years old.

An overwhelming majority of the individuals (n=277, 68.7%) knew what e-cigarettes were and more than half of these individuals (n=144, 52%) learned about e-cigarettes from friends while about one-third of them (n=101, 36.5%) found out through the internet. We also found that majority of individuals did not know about the various ingredients in e-cigarette smoke (n=225, 55.8%), neither did they know about the different levels of nicotine (n=335, 83.1%).

Almost half of the participants (n=190, 47.1%), said that people started smoking e-cigarettes due to peer pressure and to quit smoking regular cigarettes. The majority of the participants (n=238, 59.1%) did not believe that people started smoking to “fit in” or “feel cool”. About one-third of the individuals (n=129, 32%) strongly agreed that people harm themselves when smoking e-cigarettes and that they are equally harmful and equally addictive when compared to regular cigarettes. While more than half of the participants (n=203, 50.4%) had no idea how effective e-cigarettes were in helping people quit smoking regular cigarettes. Most people (n=350, 86.8%) strongly agreed or agreed with all tobacco products being dangerous with about two-thirds (n=277, 68.7%) of the participants saying that it was either easy or very easy to buy e-cigarettes.

The majority (n=239, 59.3%) stated that they “definitely will not” smoke e-cigarettes even if a good friend of theirs asked them to do so, however, strikingly, more than two-thirds (n=275, 68.2%) of them said if they did try it, they would do it without their guardian’s permission. Furthermore, about one-third (n=122, 30.3%) of the individuals stated living or being surrounded with people who use and promote the use of e-cigarettes while one-fourth of them (n=96, 23.8%) admitted to having tried one or two puffs. In addition to this, the majority (n=370, 91.8%) denied being current e-cigarette smokers and said they would not recommend or promote the use of e-cigarettes to others. The results of the various questions as percentages of the whole sample population, when compared with gender and grade of the individual have been presented in Table 1 and Table 2 respectively.

**Table 1:** Table representing the knowledge, attitudes and practices compared with gender

		Gender		P-Values
		Male Female		
Do you know what e-cigarettes are?	Yes	122(30.3%)	25(6.2%)	<0.001*
	No	155(38.5%)	101(25.1%)	
If yes, then how did you learn about them?	Internet	41(10.2%)	2(0.5%)	<0.001*
	Friends	60(14.9%)	5(1.2%)	
	Television	70(17.4%)	1(0.2%)	
	Magazines	74(18.4%)	5(1.2%)	
	Newspapers	8(2%)	25(6.2%)	
	Did not know about e-cigarettes	11(2.7%)	101(25.1%)	
Are you aware of the various ingredients and chemicals in e-cigarette smoke?	Yes	78(19.4%)	100(24.8%)	0.006*
	No	69(17.1%)	156(38.7%)	
Do you know about the different levels of nicotine in e-cigarettes?	Yes	36(8.9%)	111(27.5%)	0.002*
	No	32(7.9%)	224(55.6%)	
What do you think makes people start smoking e-cigarettes?	Stress	19(4.7%)	5(1.2%)	<0.001*
	Depression	33(8.2%)	11(2.7%)	
	Peer pressure	10(2.5%)	35(8.7%)	
	Acceptability in family	58(14.4%)	42(10.4%)	
	Recreational use	24(6%)	54(13.4%)	
	To quit smoking regular cigarettes	69(17.1%)	43(10.7%)	
How effective do you think e-cigarettes are in helping people quit smoking regular cigarettes?	Extremely effective	20(5%)	32(7.9%)	0.062
	Slightly effective	42(10.4%)	61(15.1%)	
	No idea	62(15.4%)	141(35%)	
	Slightly ineffective	7(1.7%)	10(2.5%)	
	Extremely ineffective	16(4%)	12(3%)	
What do you think about the statement “people harm themselves when smoking e-cigarettes”?	Strongly agree	43(10.7%)	90(22.3%)	0.456
	Agree	50(12.4%)	78(19.4%)	
	Undecided	34(8.4%)	51(12.7%)	
	Disagree	12(3%)	13(3.2%)	
	Strongly disagree	8(2%)	24(6%)	
When comparing harmful effects of e-cigarettes to regular cigarettes, how harmful do you think they are?	Much more harmful	27(6.7%)	45(11.2%)	<0.001*
	Slightly more harmful	12(3%)	30(7.4%)	
	Equally harmful	31(7.7%)	109(27%)	
	Slightly less harmful	46(11.4%)	54(13.41%)	
	Much less harmful	31(7.7%)	18(4.5%)	
When comparing the addiction of e-cigarettes to regular cigarettes, how addictive do you think they are?	Much more addictive	30(7.4%)	74(18.4%)	0.061
	Slightly more addictive	17(4.2%)	23(5.7%)	
	Equally addictive	46(11.4%)	83(20.6%)	
	Slightly less addictive	33(8.2%)	52(12.9%)	
	Much less addictive	21(5.2%)	24(6%)	
What do you think about the statement “all tobacco products are dangerous”?	Strongly agree	74(18.4%)	162(40.2%)	0.028*
	Agree	52(12.9%)	62(15.4%)	
	Undecided	12(3%)	13(3.2%)	
	Disagree	7(1.7%)	10(2.5%)	
	Strongly disagree	2(0.5%)	9(2.2%)	
Do you think smoking e-cigarettes makes people young people “fit in”, feel “cool” and become socially more acceptable?	Yes	57(14.1%)	108(26.8%)	0.503
	No	90(22.3%)	148(36.7%)	
How easy do you think it is to buy e-cigarettes for young people of your age?	Very easy	60(14.9%)	69(17.1%)	0.004*
	Easy	53(13.2%)	95(23.6%)	
	Difficult	21(5.2%)	76(18.9%)	
	Very difficult	13(3.2%)	16(4%)	
If a good friend of yours wanted you to try e-cigarettes, would you try them?	Definitely will	27(6.7%)	18(4.5%)	<0.001*
	Probably will	37(9.2%)	34(8.4%)	
	Probably will not	22(5.5%)	26(6.5%)	
	Definitely will not	61(15.1%)	178(44.2%)	
If you ever try smoking e-cigarettes, do you think you would do it with your guardian’s permission?	Yes	40(9.9%)	107(26.6%)	0.137
	No	40(9.9%)	88(21.8%)	
Are you surrounded by, or live with people who use and promote the use of e-cigarettes?	Yes	50(12.4%)	72(17.9%)	0.216
	No	97(24.1%)	184(45.7%)	
Do you, or have you ever smoked e-cigarettes? (Even one or two puffs.)	Yes	60(14.9%)	36(8.9%)	<0.001*
	No	87(21.6%)	220(54.6%)	
Are you a current e-cigarette smoker? (Smoked in the past 30 days.)	Yes	22(5.5%)	11(2.7%)	<0.001*
	No	125(31%)	245(60.8%)	
Would you ever promote or recommend the use of e-cigarettes to other people if you got the chance to?	Yes	20(5%)	12(3%)	0.001
	No	127(31.5%)	244(60.5%)	

**Table 2:** Table representing the knowledge, attitudes and practices compared with level of schooling

		Grade			P-Values
		Middle School	High School	Not enrolled	
Do you know what e-cigarettes are?	Yes	64(15.9%)	170(42.2%)	43(10.7%)	<0.001*
	No	78(19.4%)	42(10.4%)	6(1.5%)	
If yes, then how did you learn about them?	Internet	21(5.2%)	61(15.1%)	19(4.7%)	<0.001*
	Friends	28(6.9%)	96(23.8%)	20(5%)	
	Television	9(2.2%)	10(2.5%)	0(0%)	
	Magazines	3(0.7%)	2(0.5%)	2(0.5%)	
	Newspapers	3(0.7%)	1(0.2%)	2(0.5%)	
	Did not know about e-cigarettes	78(19.4%)	42(10.4%)	6(1.5%)	
Are you aware of the various ingredients and chemicals in e-cigarette smoke?	Yes	42(10.4%)	105(26.1%)	31(7.7%)	<0.001*
	No	100(24.8%)	107(26.6%)	18(4.5%)	
Do you know about the different levels of nicotine in e-cigarettes?	Yes	15(3.7%)	42(10.4%)	11(2.7%)	0.02*
	No	127(31.5%)	170(42.2%)	38(9.4%)	
What do you think makes people start smoking e-cigarettes?	Stress	13(3.2%)	35(8.7%)	4(1%)	<0.001*
	Depression	22(5.5%)	38(9.4%)	8(2%)	
	Peer pressure	61(15.1%)	28(6.9%)	4(1%)	
	Acceptability in family	6(1.5%)	7(1.7%)	3(0.7%)	
	Recreational use	22((5.5%)	41(10.2%)	14(3.5%)	
	To quit smoking regular cigarettes	18(4.5%)	63(15.6%)	16(4%)	
How effective do you think e-cigarettes are in helping people quit smoking regular cigarettes?	Extremely effective	34(8.4%)	13(3.2%)	5(1.2%)	0.001*
	Slightly effective	34(8.4%)	56(13.9%)	13(3.2%)	
	No idea	60(14.9%)	116(28.8%)	27(6.7%)	
	Slightly ineffective	5(1.2%)	12(3%)	0(0%)	
	Extremely ineffective	9(2.2%)	15(3.7%)	4(1%)	
What do you think about the statement “people harm themselves when smoking e-cigarettes”?	Strongly agree	74(18.4%)	48(11.9%)	11(2.7%)	<0.001*
	Agree	33(8.2%)	73(18.1%)	22(5.5%)	
	Undecided	10(2.5%)	63(15.6%)	12(3%)	
	Disagree	8(2%)	13(3.2%)	4(1%)	
	Strongly disagree	17(4.2%)	15(3.7%)	0(0%)	
When comparing harmful effects of e-cigarettes to regular cigarettes, how harmful do you think they are?	Much more harmful	35(8.7%)	29(7.2%)	8(2%)	0.003*
	Slightly more harmful	16(4%)	23(5.7%)	3(0.7%)	
	Equally harmful	55(13.6%)	72(17.9%)	13(3.2%)	
	Slightly less harmful	19(4.7%)	64(15.9%)	17(4.2%)	
	Much less harmful	17(4.2%)	24(6%)	8(2%)	
When comparing the addiction of e-cigarettes to regular cigarettes, how addictive do you think they are?	Much more addictive	71(17.6%)	28(6.9%)	5(1.2%)	<0.001*
	Slightly more addictive	14(3.5%)	22(5.5%)	4(1%)	
	Equally addictive	16(4%)	85(21.1%)	28(6.9%)	
	Slightly less addictive	25(6.2%)	52(12.9%)	8(2%)	
	Much less addictive	16(4%)	25(6.2%)	4(1%)	
What do you think about the statement “all tobacco products are dangerous”?	Strongly agree	101(25.1%)	112(27.8%)	23(5.7%)	0.002*
	Agree	27(6.7%)	69(17.1%)	18(4.5%)	
	Undecided	3(0.7%)	18(4.5%)	4(1%)	
	Disagree	4(1%)	9(2.2%)	4(1%)	
	Strongly disagree	7(1.7%)	4(1%)	0(0%)	
Do you think smoking e-cigarettes makes people young people “fit in”, feel “cool” and become socially more acceptable?	Yes	63(15.6%)	83(20.6%)	19(4.7%)	0.329
	No	79(19.6%)	129(32%)	30(7.4%)	
How easy do you think it is to buy e-cigarettes for young people of your age?	Very easy	38(9.4%)	77(19.1%)	14(3.5%)	<0.001*
	Easy	29(7.2%)	91(22.6%)	28(6.9%)	
	Difficult	52(12.9%)	38(9.4%)	7(1.7%)	
	Very difficult	23(5.7%)	6(1.5%)	0(0%)	
If a good friend of yours wanted you to try e-cigarettes, would you try them?	Definitely will	21(5.2%)	22(5.5%)	2(0.5%)	0.015*
	Probably will	13(3.2%)	44(10.9%)	14(3.5%)	
	Probably will not	8(2%)	34(8.4%)	6(1.5%)	
	Definitely will not	100(24.8%)	112(27.8%)	27(6.7%)	
If you ever try smoking e-cigarettes, do you think you would do it with your guardian’s permission?	Yes	57(14.1%)	52(12.9%)	19(4.7%)	0.002*
	No	85(21.1%)	160(39.7%)	30(7.4%)	
Are you surrounded by, or live with people who use and promote the use of e-cigarettes?	Yes	59(14.6%)	51(12.7%)	12(3%)	<0.001*
	No	83(20.6%)	161(40%)	37(9.2%)	
Do you, or have you ever smoked e-cigarettes? (Even one or two puffs.)	Yes	30(7.4%)	57(14.1%)	9(2.2%)	0.217
	No	112(27.8%)	155(38.5%)	40(9.9%)	
Are you a current e-cigarette smoker? (Smoked in the past 30 days.)	Yes	17(4.2%)	15(3.7%)	1(0.2%)	0.115
	No	125(31%)	197(48.9%)	48(11.9%)	
Would you ever promote or recommend the use of e-cigarettes to other people if you got the chance to?	Yes	11(2.7%)	18(4.5%)	3(0.7%)	0.802
	No	131(32.5%)	194(48.1%)	46(11.4%)	

Males significantly knew more about what e-cigarettes were (p <0.001), different ingredients present in their smoke (p=0.006) and different levels of nicotine (p=0.002). Whereas more females chose the option I do not know about e-cigarettes (p <0.001) and thought depression was the cause of e-cigarette smoking (p <0.001). Furthermore, statistically significant differences were also found when comparing gender, if the individuals had ever tried smoking e-cigarettes (p <0.001), were they current e-cigarette smokers (p <0.001) and if they would promote or recommend e-cigarette use (p=0.001). More females answered no to the aforementioned questions. Significantly more females also thought that e-cigarettes were equally harmful as regular cigarettes (p <0.001), strongly agreed with tobacco products being dangerous (p=0.028), thought it was difficult to buy e-cigarettes (p=0.004) and that they definitely would not try e-cigarettes if a good friend of their asked them to (p <0.001).

In addition to this, when the level of schooling was compared with the responses, statistically significant differences were found. High school students knew more about what e-cigarettes were (p <0.001), learned about e-cigarettes through their friends (p <0.001), were more aware of its ingredients (p <0.001) and did not know about its different levels of nicotine (p=0.02). When their attitudes were compared with level of schooling, more middle school students thought: peer pressure was the cause of e-cigarette smoking (p <0.001), e-cigarettes were extremely effective in helping people quit regular cigarettes (p=0.001), people were harming themselves by smoking e-cigarettes (p <0.001), e-cigarettes were much more harmful (p=0.003) and much more addictive (p=0.002) than regular cigarettes. Furthermore, more middle school students strongly agreed with all tobacco products being dangerous (p=0.001), thought it was either difficult or very difficult to buy e-cigarettes (p <0.001), said they definitely would not try e-cigarettes if a good friend of theirs’ asked them too (p=0.015), said that they would try it with their guardian’s permission (p=0.002) and that they either lived with, or were surrounded by, people who use and promote the use of e-cigarettes (p= <0.001).

## Discussion

Our principal finding was that more than half of the teenagers did not have any significant knowledge about e-cigarettes and showed a restraining attitude towards its use. On the contrary, previous studies have shown that increased advertising and branding about e-cigarettes has markedly contributed to a rapid rise in e-cigarette consumption among high school students [12]. An analogous finding was laid forward in a cross-sectional study conducted in England [13] which concluded that teenagers use e-cigarettes to experiment with the product rather than to utilize it for its actual intended purpose, that is, to quit smoking. This deviation from the global norm is perhaps due to a lack of e-cigarette advertising and branding in Pakistan as well as strong cultural and familial beliefs that stigmatize smoking and the use of devices that simulate smoking due to its potentially harmful effects on health.

Secondly, we found statistical differences in the knowledge, attitude, and practices between males and females with males having a more positive outlook towards e-cigarettes with much more of them being current users as concluded by a previous study [14]. This is perhaps due to males being much less likely to be judged on their views and practices as compared to females owing to strong patriarchal beliefs which are deeply embedded in Pakistani culture [15]. This could be further explained by the fact that such social practices by females are considered a taboo in Pakistan.

We also found that an overwhelming majority of e-cigarette users held beliefs consistent with the claims made in e-cigarette advertisements; people showed a significantly positive attitude towards the use of e-cigarettes considering it a less dangerous, and hence, a better alternative to conventional cigarettes which is consistent with the results of previous studies [16-20]. The motivation for using e-cigarettes also came from the thought that these could help them avoid regular cigarette smoking which was also concluded in recent studies [21- 23]. Furthermore, those participants who were not currently enrolled in any school also believed that people smoke e-cigarettes to stop smoking regular cigarettes and they also believed that e-cigarettes were less harmful than regular cigarettes, hence, proving that the reduced harms perception of e-cigarettes was associated with increased likelihood of usage of e-cigarettes, as is consistent with another study [24]. Furthermore, most participants stated that their families were not supportive of e-cigarette use, that if they were to smoke e-cigarettes they would do so without their guardian’s permission and that they would not promote the use of e-cigarettes. Hence, showing that the social acceptability of e-cigarettes is low and that they are considered harmful by a vast majority of the population. The majority also believed that smoking e-cigarettes did not make one look cool or trendy and that all tobacco products are equally dangerous. This reasoning could be responsible for their reduced willingness to try e-cigarettes. Quite a few of the participants also thought that e-cigarettes were equally addictive and had no idea about the effectiveness of e-cigarettes in order to quit smoking regular cigarettes. This could be because most of the participants did not know anyone who was using e-cigarettes to quit smoking regular cigarettes or that they did not know anyone who was successful in their attempt to do so. The reduced knowledge about e-cigarettes could also be because of the novelty of e-cigarettes and the lack of data available on it.

Since the study was limited to the city of Karachi, further studies should be conducted in other areas of Pakistan which may cover lower socioeconomic rural regions and people of different age groups. The side effects reported by the beginners compared with the benefits that the experienced people felt when smoking e-cigarettes should be included in the upcoming studies. A check-and-balance system, to see if the people who wanted to start e-cigarette use as a means to quit conventional cigarette smoking have actually brought this change in their behavior. It is also recommended that further investigation on e-cigarettes takes place focusing mainly on its health risks or benefits and its long-term effects. Moreover, further research to determine prevalence rates among various demographic groups, as well as in different cities should also be conducted. The impact of e-cigarette promotion and advertising on e-cigarette use among youths should also be evaluated, in order to be able to better understand the increasing use of e-cigarettes.

Having said that, our study had some limitations which need to be considered. Since only teenagers in Karachi were specifically recruited in the study, diverse perspectives were not gathered as the population did not include teenagers from different geographic areas and socioeconomic statuses. Furthermore, most of the participants were middle or high school students leaving a small minority which was not enrolled in any school. The study did not highlight the side effects of using e-cigarettes that the users came across as beginners. Also, no proof of the direct association between the people experiencing benefits by the use of e-cigarettes could be provided by this study. The interviewees who were current e-cigarette users and were working towards quitting the use of these could not be identified. Also, we could not find if the participants considering the use of e-cigarettes as a means of quitting conventional cigarette smoking actually succeeded in quitting. Finally, long-term smokers who had quit were not taken into account and could not be compared with current smokers.

## Conclusions

This study was carried out to find out the knowledge and attitude of teenagers towards electronic cigarettes and according to our results, it was evident that though a majority of population had a basic idea about electronic cigarettes, there was lack of proper knowledge along with negative attitudes towards e-cigarette use among teenagers in Pakistan due to cultural and social stigmas and lack of advertising. Also, it was observed that males and females had considerable differences in their opinions regarding e-cigarettes use owing to such social practices being considered taboo by females and males having greater freedom due to patriarchal, familial and cultural systems.
